# Human Erythropoietin Effect in Postoperative Visual Loss Following Spine Surgery: A Case Report

**DOI:** 10.5812/aapm.7291

**Published:** 2014-04-06

**Authors:** Valiollah Hassani, Mohammad Mohsen Homaei, Ali Shahbazi, Mohammad Mahdi Zamani, Saeid Safari, Shermila Nadi, Abolfazl Rahimizadeh, Mohammad Hossein Lashkari, Siamak Alizadeh zendehrood

**Affiliations:** 1 Minimally Invasive Surgery Research Center, Iran University of Medical Sciences, Tehran, Iran; 2 Department of Anesthesiology, Rasoul Akram Medical Center, Iran University of Medical Sciences, Tehran, Iran; 3 Physiology Research Center (PRC), Iran University of Medical Sciences, Tehran, Iran; 4 Department of Neurosurgery, Pars General Hospital, Tehran, Iran; 5 Department of Ophthalmology, Iran University of Medical Sciences, Tehran, Iran

**Keywords:** Complications, Optic Neuropathy, Ischemic, Postoperative Period, Postoperative, Spinal Cord Ischemia, Vision Disorders

## Abstract

**Introduction:**

Postoperative visual loss (POVL) has become the focus of attention for anesthesiologists as a hallmark of perioperative management in spine surgery. A number of Intraoperative and postoperative factors has been documented but the exact etiology is still unclear. Nowadays, perioperative management and also complete curing of POLV is a big question of ophthalmologists and anesthesiologists. The purpose of this case report is to present a unique experience of complete curing the POLV.

**Case Presentation:**

Our patient was a 61-year-old man, with 75 kg weight and 180 cm height. The patient had no history of visual impairment except mild cataract in his right eye. The patient had a history of diffuse idiopathic skeletal hyperostosis (DISH). The patient had undergone lumbar surgery in prone position. The operation time was about 6 hours. About 30 minutes after transferring to postanesthesia care unit (PACU), patient was awake and complained of losing his eyesight. There was no vision and light perception in his right eye on primary examination. Urgent ophthalmologist consultation was requested. In ophthalmology examinations, the pupil reflex to light was absent in the right eye. After obtaining patients and his family informed consent, four hours after the operation, 40000 I.U. of recombinant human erythropoietin (rhEPO) was administered for patient in PACU (IV infusion, in 30 min). An ophthalmologist visited him every 6 hours after administration of rhEPO. The patient was transferred to intensive care unit (ICU) one hour later with total visual loss in the right eye. Ophthalmologic examination after the second dose of rhEPO, 30 hours after the operation, reported pupil reflex enhancement and light perception in his right eye. Finally the third dose of rhEPO (40000 I.U., IV infusion) was administered on the third day. Ophthalmologic examination after the third dose of rhEPO, 60 hours after the operation, reported normal pupillary light reflex of the right eye and visual acuity improvement to 20/20. The patient was discharged from hospital after six days, with normal visual acuity and without any new complications except surgical site pain.

**Conclusions:**

Our case report showed the therapeutic effect of rhEPO in complete curing of POVL. Regarding the side effects of EPO such as thrombogenic effects or mild hemodynamic changes like transient sinus tachycardia during infusion, it seems that beneficial effects of EPO is more than its disadvantages and expenses, for patients with POVL.

## 1. Introduction

Postoperative visual loss (POVL) is a rare but important complication in non-ocular surgeries (1, 2). This complication has been reported following several kinds of surgeries, including, spinal, cardiac, vascular, brain, sinus and even prostatectomy surgeries (3, 4). Many studies have reported POVL in spinal surgeries, in prone positioning (5, 6). Due to malpositioning or head movements in prone position, external pressure to eyes increases in spinal surgery and results in POVL (7). In addition to positioning, some preoperational conditions were reported as probable etiologies of POVL, such as previous history of endothelial vascular disturbances (hypertension, diabetes mellitus, smoking, atherosclerosis), anemia and closed angle glaucoma; and some operational risk factors were reported also, including severe hemorrhage, and hypotension during the operation (4).

It has been documented that central retinal artery occlusion (CRAO) and ischemic optic neuropathy (ION) are the common mechanisms of POVL (8). Symptoms of POVL, differ in patients, depending on the location and extent of the lesion; but loss of visual acuity in entire or in a part, lack of light perception, and absence of pupillary light reflex are the common symptoms (9). In POVL, normal fundoscopy with impaired ocular movement due to extra-ocular muscle oedema may be seen (10). Abnormal fundoscopic findings are more probable in anterior ischemic optic neuropathy (AION) and CRAO comparing posterior ischemic optic neuropathy (PION) (7).

Although some perioperative consideration such as correction of anemia, regulating blood pressure, lowering the ocular or optic nerve pressure, and administration of acetazolamide and mannitol have been suggested, there is no reliable therapeutic option nowadays, and some of the patients would lose their vision for lifetime (4, 9, 11).

The exact pathophysiological mechanism of ION is not known. Some animal studies have been developed to clarify the pathophysiologic mechanisms of ION and effects of potential therapeutic agents (9, 12). Recently, some studies have shown the role of erythropoietin and its receptors in the pathophysiology of ION (13); and some animal studies demonstrated that recombinant human erythropoietin (rhEPO) can reverse the histopathological process of ION and causes functional recovery following ION (14, 15). Here we present a case of POVL following the PSF surgery, treated successfully with rhEPO.

## 2. Case Presentation

The authors obtained patient and his family’s consent and consulted the institutional ethics review board (IRB) for approval (not deemed necessary by the IRB) for publishing in this journal. The patient was a 61 years old man, 75 kg weight and 180 cm height. He was admitted to Pars General Hospital, Tehran, Iran, for posterior spinal fusion (PSF) of C7-T1. He had a history of neck trauma about one year before. In past medical history, there was no history of hypertension, diabetes mellitus or, ischemic heart disease. He had no history of visual impairment, except mild cataract in his right eye. He had a history of diffuse idiopathic skeletal hyperostosis (DISH). Before the operation, hemoglobin was 13.15 g/dL; platelet count was 235×10 3/µL, and clotting time (CT) and bleeding time (BT) were in the normal range. Biochemistry tests did not show any pathologic states. Preoperative consultation of anesthesiologist, revealed no risk factor for general anesthesia. The electrocardiogram (ECG) had normal findings with normal sinus rhythm without any ST segment or T wave changes. He was transferred to the operating room to undergo PSF with general anesthesia. 

Anesthesia pre-medications were 2 mg midazolam, 30 μg sufentanil and 100 mg lidocaine. Anesthesia was induced by 300 mg Thiopental-Na and 40 mg atracurium. Patient was intubated with a number eight endotracheal tube and positioned in prone position. Patient head positioned on a horseshoe head rest. Anesthesia was maintained during the operation by using propofol infusion (75-100 μg/kg/min), in conjunction with a remifentanil infusion (0.1-0.2 μg/kg/min), atracurium (0.2 mg/kg/every 30 min) and a mixture of nitrous oxide (3 L/min) and oxygen (3 L/min). The ventilation protocol consisted of an inspired oxygen fraction of 1.0, inspiratory to expiratory ratio of 1:2, and a respiratory rate adjusted to normocapnia (end-tidal carbon dioxide partial pressure between 30 and 40 mmHg). Mechanical ventilation was performed with a tidal volume of 10 mL/kg ideal body weight (IBW) and ZEEP (zero-positive end expiratory pressure).

The operation time was about 6 hours. Standard monitoring included continuous ECG, pulseoximetry, capnography and urine output during anesthesia. Noninvasive BP measurements were performed at 5-min intervals. Urine output was 800 mL during the operation and there was no significant change in blood pressure. Blood loss was 2500 mL during the operation, which was replaced by 900 mL packed red blood cell, 200 mL fresh frozen plasma and 3000 mL of crystalloids. The patient was extubated after reversing with 3mg neostigmine and 1.5 mg atropine sulfate.

About thirty minutes after transferring to postanesthesia care unit (PACU), patient was awaked and complained ofvisual impairment in his right eye. There was no vision and light perception in his right eyeinprimary examinations. Urgent ophthalmologist consultation was requested. In ophthalmology examinations, left eye had normal visual acuity, normal pupil reflex and normal fundoscopy. In the right eye, the pupil reflex to light was absent and there was no light perception, and positive Marcus Gunn sign, and mild cataract was identified. Right eye fundoscopy showed normal appearance of retina and optic disk with no abnormality in retinal vessels. PION was proposed as diagnosis. Postoperative hemoglobin was 10 mg/dL, while other laboratory tests including arterial blood gases had normal findings. Postoperative ECG and chest X-ray did not have any pathologic changes.

Finally, four hours after the operation, 40000 I.U. of rhEPO (PD poein®, Pooyesh Darou, Tehran, Iran) was administered (IV infusion, 30 min) to patient in postanesthesia care unit (PACU). An ophthalmologist visited the patient every 6 hours until complete curing of visual loss. The patient was transferred to intensive care unit (ICU) one hour later with total visual loss in his right eye. Patient received three doses of methylprednisolone (500 mg, IV, every 8 hours) and one dose of enoxaparin (60 mg, S.C.) in ICU. Thirty hours after the operation, the second dose of rhEPO (40000 I.U., IV infusion) was administered. Sixth ophthalmologic examination was performed after the second dose of rhEPO and after 30 hours from the end of operation, which revealed pupil reflex enhancement and light perception in the right eye, for the first time. In eighth ophthalmologist examination, after the second dose of rhEPO visual acuity was improved to 10/20, eighth ophthalmologist examination was performed after 42 hours after the operation.

Finally, the third dose of rhEPO (40000 I.U., IV infusion) was administered on the third day (52 hours after the operation). In 11th ophthalmologist visit, after the third dose of rhEPO, pupillary reflex of the right eye had normal findings, and visual acuity gradually progressed to 20/20. Eleventh ophthalmologist visit was performed after 8 hours of the third dose of rhEPO and 60 hours after the operation. The patient was discharged from hospital after six days, with normal visual acuity and without any new complications except surgical site pain. No adverse effects were seen after rhEPO infusion, except transient sinus tachycardia during infusion. After six months of follow up, his bilateral visual acuity was performed, and his visual acuity remained 20/20 after 6 months of the operation.

## 3. Discussion

This case report introduced a patient with POVL in one eye, after spinal surgery in prone positioning. Previous reports have shown that this complication is more frequent after spinal surgery compared to other surgeries such as cardiac and vascular surgeries (16, 17). Based on the American Society of Anesthesiology postoperative visual loss registry report, 83 cases from all 93 cases of POVL were occurred following spinal surgery, and most of these patients had prone positioning during the operation. POVL can also occur without external pressure to eyes in prone positioning (18). Duration of surgery equal or more than six hoursis reported as another risk factor predisposing POVL (18). Duration of surgery in this case was 6 hours approximately, which may have contributed to visual loss in this patient. Another predisposing factor in this patient was considerable hemorrhage (about 2500 mL). Most of POVL cases had massive hemorrhage during the operation (18). Although the hemoglobin concentration in this patient was 10 mg/dL at the end of operation, but it seems that this index is not reliable especially after fluid replacement therapy. Hemodilution was also reported to have a role in developing POVL (9); However, senile atherosclerotic changes was not present in our patients as another known risk factors of POVL.

CRAO and anterior or posterior ION are the main mechanisms of POVL (8, 9). Diagnosis is based on fundoscopic and pupillary light reflex examinations. Abnormal findings in fundoscopy such as pale ischemic retina with cherry-red spot or edematous disc with or without prepapillary flame-shaped hemorrhages have been seen in CARO and AION cases, respectively, while PION cases had normal fundoscopic examination (7). Total visual and light perception loss, lack of pupillary light reflex and positive Marcus Gunn pupil in the presence of normal fundoscopy, implemented the diagnosis of PION in this case. However, reports of the American Society of Anesthesiologists (ASA) indicated that POVL due to PION is more likely to involve both eyes (7).

There is not a reliable therapeutic option for POVL patients at present, and considerable number of patients would lose their visual acuity for entire lifetime (11, 12). Based on the findings of animal models showing the beneficial effects of rhEPO on ION, we decided to treat this case with rhEPO (13-15). It was seen that rhEPO (40,000 I.U., in three consecutive days) completely reversed the POVL in our patient.

It has been shown that erythropoietin (EPO) can protect neural cells from injuries induced by hypoxia, infections and neurotoxicity (19-21). EPO released during hypoxia can inhibit neural cell death by its antiapoptotic mechanisms (22) and also can promote neurogenesis in the central nervous system (23). EPO receptors are normally expressed in retinal ganglion cells and may have physiological roles in the retina (24, 25). EPO can prevent apoptosis in animal studies, via activation of some protein kinases pathways and recruitment of antiapoptotic molecules like NF-Ƙβ and bcl2 in the retina (14, 15, 21). The important finding of this study was that rhEPO reversed total visual loss, completely after 60 hours. The rhEPO possibly via its antiapoptotic mechanisms cured our patients with monocular POVL. Of course, in a recent study by Quraishi et al. a single patient following spinal surgery,was presented with transient bilateral POVL. This patient’s visual loss improved within 48 hours (26). POLV was monocular in our patients and was improved 30 hours from the operation and 24 hours after the first dose of rhEPO. According to Quraishi report, our result might not be interfered by rhEPO therapy,nonetheless the first time of improvement occurred sooner than other reports, may support the beneficial effects of rhEPO. On the other hand, our case of monocular POVL, reversed completely, exact 8 hours after the third dosage of rhEPO and 60 hours after the operation. Evidently, we have not continuous ophthalmologist visit, but in 8th visit of ophthalmologist, 14 hours after the second dose of rhEPO, visual acuity was 10/20, and 8 hours after the third dose of rhEPO, visual acuity reversed completely to 20/20.

In conclusion, this case report showed the beneficial effect of rhEPO in complete curing of POVL. Regarding a few side effects of EPO such as thrombogenic effects or mild hemodynamic changes like transient sinus tachycardia during infusion, it seems that beneficial effects of EPO is more than its disadvantages and expenses, for patients with POVL. However, further investigations need to consider rhEPO as a therapeutic option in patients with postoperative visual loss to confirm its beneficial effects. This finding is promisingin the absence of specific therapeutic option for POVL.
